# The Dutch Hip Fracture Audit: evaluation of the quality of multidisciplinary hip fracture care in the Netherlands

**DOI:** 10.1007/s11657-019-0576-3

**Published:** 2019-03-01

**Authors:** Stijn C. Voeten, Arend J. Arends, Michel W. J. M. Wouters, Bastiaan J. Blom, Martin J. Heetveld, Monique S. Slee-Valentijn, Pieta Krijnen, Inger B. Schipper, J. H. (Han) Hegeman, On behalf of the Dutch Hip Fracture Audit (DHFA) Group, A. J. Arends, B. J. Blom, M. van Eijk, M. J. Heetveld, J. H. Hegeman, R. M. Houwert, M. C. Luyten, B. G. Schutte, M. S. Slee-Valentijn, S. C. Voeten

**Affiliations:** 1 Department of Trauma Surgery, Leiden University Medical Center, Albinusdreef 2, Leiden, 2333ZA The Netherlands; 2 Dutch Institute for Clinical Auditing, Leiden, The Netherlands; 3 Maasstadziekenhuis, Rotterdam, The Netherlands and Dutch Geriatric Society (NVKG), Utrecht, The Netherlands; 4 Department of Surgery, Netherlands Cancer Institute-Antoni van Leeuwenhoek Hospital, Amsterdam, The Netherlands; 5 Department of Orthopedics, Flevoziekenhuis, Almere, The Netherlands; 6 Department of Surgery, Spaarne Gasthuis, Haarlem-Hoofddorp, The Netherlands; 7 Department of Geriatric Rehabilitation, Cordaan, Amsterdam and The Dutch Society of Internal Medicine, The Netherlands; 8 Department of Trauma Surgery, Ziekenhuisgroep Twente, Almelo-Hengelo, The Netherlands

**Keywords:** Audit, Benchmark, Hip fracture, Quality indicators, Dutch Hip Fracture Audit

## Abstract

***Summary*:**

The nationwide Dutch Hip Fracture Audit (DHFA) is initiated to improve the quality of hip fracture care by providing insight into the actual quality of hip fracture care in daily practice. The baseline results demonstrate variance in practice, providing potential starting points to improve the quality of care.

**Purpose:**

The aim of this study is to describe the development and initiation of the DHFA. The secondary aim is to describe the hip fracture care in the Netherlands at the start of the audit and to assess whether there are differences in processes at baseline between hospitals.

**Methods:**

Eighty-one hospitals were asked to register their consecutive hip fracture patients since April 2016. In 2017, the first full calendar year, the case ascertainment was determined at audit level. Three quality indicators were used to describe and assess the care process at audit and hospital level: the proportion of completed variables at discharge and at 3 months after operation, time to surgery and orthogeriatric management.

**Results:**

Sixty (74%) hospitals documented 14,274 patients in the DHFA by December 2017. In 2017, the case ascertainment was 58% and the average proportion of completed variables was 77%: 91% at discharge and 30% at 3 months. The median time to operation was 18 h (IQR 7–23) for American Society of Anesthesiologists score (ASA) 1–2 patients and 21 h (IQR 13–27) for ASA 3–4 patients. Of patients aged 70 years and older, 78% received orthogeriatric management. At hospital level, all three indicators showed significant practice variance.

**Conclusion:**

Not all hospitals participate in the DHFA, and the data gathering process needs to be further optimized. However, the baseline results demonstrate an apparent variance in hip fracture practice between hospitals in the Netherlands, providing potential starting points to improve the quality of hip fracture care.

## Introduction

Clinical audits or registries of processes and outcomes of care have proven useful to improve the quality of care [1, 2]. The first audit for hip fracture care was established in Sweden, in 1988 [3]. Nowadays, several hip fracture audits exist [3–10]. As shown by the National Hip Fracture Database (the UK minus Scotland) and the Scottish Hip Fracture Audit (Scotland), the implementation of an audit leads to improved adherence to national guidelines, a decline in practice variance and improved patient outcomes [11–13].

In the Netherlands, optimal hip fracture care is described in two evidence-based Dutch guidelines—one updated in 2016 “Guideline Proximal Femur Fracture” and the other first published in 2016 “Guideline Multidisciplinary Treatment of Frail Elderly During Surgical Procedures” [14, 15]. The presence of a national guideline does not, however, automatically imply overall adherence [16]. The need for guideline adherence, alongside the motivation to improve overall hip fracture care in the Netherlands, led to the initiation of a nationwide clinical hip fracture audit in 2016, the Dutch Hip Fracture Audit (DHFA). The DHFA aims to improve the quality of care by providing insight into the actual quality of hip fracture care in daily practice, and based on its results, to define targeted initiatives to be launched to improve the overall quality of hip fracture care.

Simultaneously, healthcare professionals are increasingly required to provide a growing amount of information about their performance to governmental institutions. In the Netherlands, the patient data for multiple hip fracture quality indicators have to be reported to the Dutch National Healthcare Institute (DNHI) and the Health and Youth Care Inspectorate (HYCI) [17, 18]. As overall guidance is lacking, each hospital collects and calculates this data in its own way, a time-consuming procedure that may produce debatable results. Therefore, another goal of the DHFA was to enable hospitals to automatically deliver the results of these indicators to DNHI and HYCI in a uniform manner.

The aim of this study is to describe the development and initiation of the Dutch Hip Fracture Audit. The secondary aim is to describe the hip fracture care in the Netherlands at the start of the audit and to assess whether there are differences in processes at baseline between hospitals.

## Methods

### Initiation of the DHFA

The Dutch Association for Trauma Surgery took the initiative to join forces with all medical associations involved in the care for patients with hip fractures in a multidisciplinary audit for hip fracture care. The DHFA was established with funding from the Dutch Association of Medical Specialist.

The DHFA is overseen by a multidisciplinary clinical audit board in which medical associations involved in the hip fracture care process in the Netherlands are represented, including mandated members from the Dutch Association for Trauma Surgery (NVT), the Dutch Association of Surgeons (NVvH), the Dutch Orthopaedic Association (NOV), the Dutch Geriatric Society (NVKG) and the Dutch Society of Internal Medicine (NIV). The clinical audit board appointed a scientific committee, which decides on the contents of the DHFA and is responsible for the development of methodologically sound quality indicators.

The DHFA is part of the Dutch Institute for Clinical Auditing (DICA). DICA is an organization that facilitates nationwide audits in a uniform format for varying diseases [19]. It was founded in 2011 after colorectal surgeons initiated the Dutch Surgical Colorectal Audit (DSCA) [20]. At present, 22 nationwide clinical audits are facilitated by DICA [21–24].

The scientific bureau of DICA supports the scientific committee of DHFA with its expertise in clinical auditing and the methodologic issues involved. The data management unit of DICA provides a web-based feedback report to benchmark hospital performance using funnel plots.

### Development of the DHFA

The dataset items are based on recommendations made in national and international guidelines, items used in other international hip fracture audits and quality indicators. Every year the dataset items are evaluated and, whenever necessary, updated or adjusted. The dataset currently includes 45 items recorded at three different moments: at the moment of hospital discharge, 3 months after the operation and 1 year after the operation (see Table 3).

The DHFA data can be registered by authorized hospital employees (e.g. medical secretaries, data managers, nurse practitioners, physicians or medical specialists) in a secure web-based survey, but the medical specialist remains responsible for the completeness and correctness of the entered data. Owing to the quality improvement purpose of the audit, an informed consent is not needed to register the DHFA data of a hip fracture patient in the secure web-based survey. To ensure that accurate data is entered, data verification is directly done in the web-based survey: unrealistic answers or missing fields are being flagged. In addition, external data verification for a random sample of patients in each hospital will be performed every 3 years. For this purpose, an independent team of monitors will compare the source data in the electronic health records with the data entered in the web-based survey.

In line with privacy regulations in the Netherlands, only anonymized patient data are forwarded for analysis from the secure web-based DHFA survey to DICA by an independent data processor (Medical Research Data Management, MRDM) [25].

All 81 hospitals treating hip fracture patients in the Netherlands were asked to register the DHFA data of all patients admitted since 1 April 2016. The exclusion criteria were age under 18 years, pathologic fracture due to a malignant disease and peri-prosthetic fracture. The case ascertainment was determined for the first full calendar year (2017). To assess the case ascertainment, the total number of operated patients (i.e. patients recorded as having been operated) in the DHFA was compared to the number of patients registered by the DNHI. At audit level, the completeness of variables recorded at hospital discharge and 3 months after operation for patients who were still alive at that time was described for the periods April–December 2016 and January–December 2017.

### Quality indicators to assess the processes of hip fracture care at baseline

Three quality indicators were used to describe and assess the processes of hip fracture care at the start of the audit. The processes were evaluated at audit and hospital level for the calendar year 2017 (see Table 4 for the definitions).

1. Data completeness, determined as the proportion of completed variables for operated patients.
2. The median time to surgery, measured from admission to the emergency department to the start of surgery, was described for American Society of Anesthesiologists score (ASA) 1/2 and ASA 3/4 patients separately. Comparisons at hospital level included the number of ASA 1/2 and ASA 3/4 patients operated within the median time to surgery. Hospitals with > 10% of data missing on the variable time to surgery were excluded from this analysis.
3. For operated hip fracture patients older than 70 years, the presence of orthogeriatric management during admission was described. The proportion of patients with orthogeriatric treatment during admission was compared at hospital level. Hospitals having a special comprehensive orthogeriatric ward were identified. To be identified as a hospital with an orthogeriatric ward, more than 50% of the orthogeriatric care had to be provided on the special ward. Hospitals with > 10% missing on the variable orthogeriatric management were excluded from this analysis.

## Results

### Case ascertainment

A total of 14,274 patients admitted in the period April 2016–December 2017 were included in the DHFA by 60 hospitals (74%), 3188 patients in 2016 and 11,086 patients in 2017. Of the included patients, 278 (1.9%) were treated non-operative; for 341 patients (2.4%), the type of treatment was missing. One hundred and forty-eight (1.0%) patients had a second hip fracture and were entered twice in the DHFA.

The case ascertainment of the operated hip fracture patients in 2017 was 58%, as 10,612 of the 18,385 operated hip fracture patients registered at the DNHI were also registered in the DHFA.

### Data completeness

**Fig. 1:**
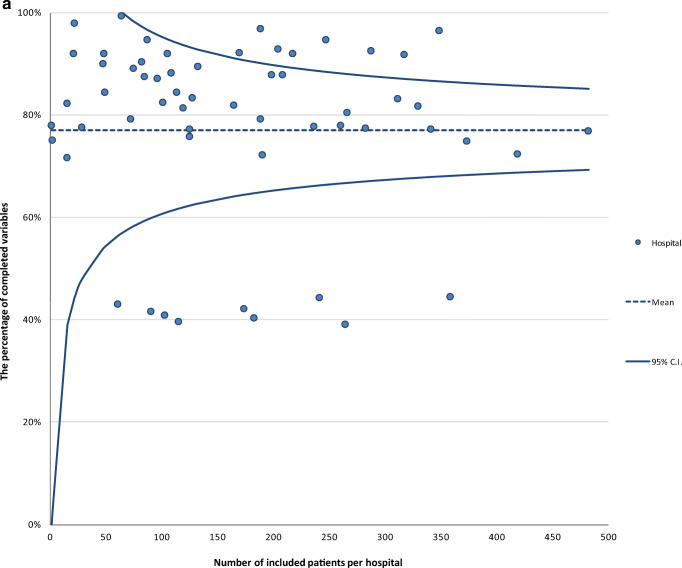

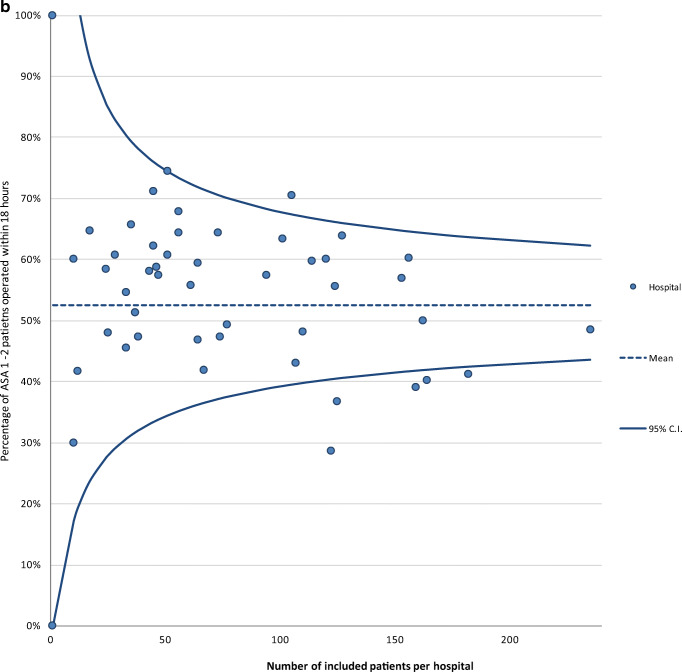

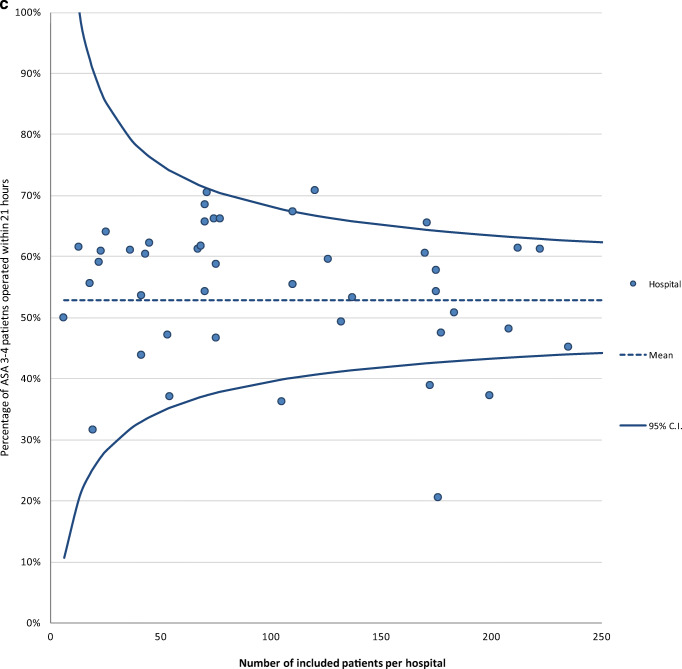

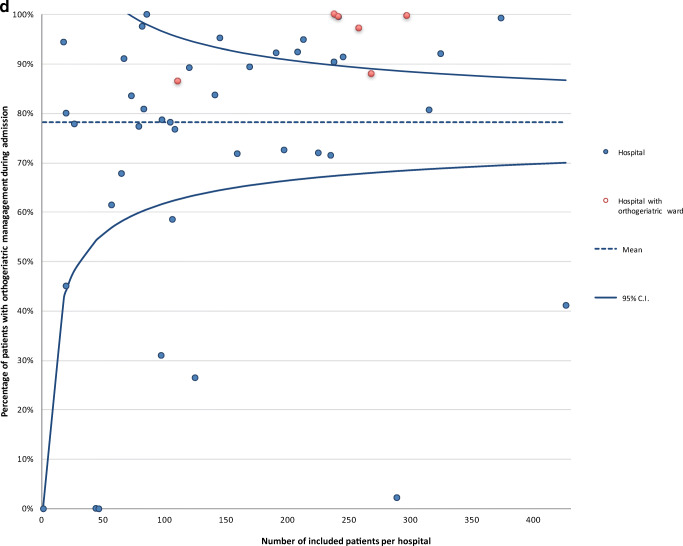
**a** Funnel plot of the proportion of variables (variables included date of birth, gender, type of fracture, type of treatment, ASA score, date and time of arrival at emergency department, date and time of surgery, consultation of geriatrician, date of discharge, type of anaesthesia, complications, Katz Index of Independence in Activities of Daily Living at admission, mobility score at admission, living situation before admission, reoperations, 3-month Katz Index of Independence in Activities of Daily Living at admission, 3-month mobility score, 3-month living situation) completed per hospital in the Dutch Hip Fracture Audit in 2017. **b** The percentage of ASA 1/2 patients operated within the nationwide median time difference in hours between admission and start of operation per hospital in 2017. The horizontal line represents the mean proportion of all patients who were operated within the median time of 18 h. Each dot represents the proportion of patients in a specific hospital who were operated within the median time. **c** The percentage of ASA 3/4 patients operated within the nationwide median time difference in hours between admission and start of operation per hospital in 2017. The horizontal line represents the mean proportion of all patients who were operated within the median time of 21 h. Each dot represents the proportion of patients in a specific hospital who were operated within the median time. **d** Orthogeriatric management during admission of patients 70 years and older with a surgically treated hip fracture.

The proportion of completed variables recorded at hospital discharge was 95% in 2016 and 91% in 2017. Average completeness of the variables recorded 3 months after operation was much lower: 37% in 2016 and 30% in 2017 (Table 1). The proportion of completed variables in 2017 was 77% at audit level and differed significantly at hospital level, ranging from 39 to 99%. For nine hospitals, data completeness was significantly lower compared to the audit average (Fig. 1a).

**Table 1:** Data completeness per variable of the clinical and 3-month section of registered patients in the DHFA

Completeness clinical section, *n* (%)	2016^∆^ *N* = 3188	2017 *N* = 11,086
Date of birth	3185 (99.9)	11,081 (100)
Gender	3183 (99.8)	11,072 (99.9)
Type of fracture	2792 (87.6)	9127 (82.3)
Type of treatment	3113 (97.6)	10,820 (97.6)
ASA score*	2763 (90.8)	9013 (84.9)
Time arrival at the ER^¶^	3013 (94.5)	10,720 (96.7)
Date of surgery*	3029 (99.5)	10,596 (99.8)
Anaesthesia type*	2841 (93.4)	9466 (85.4)
Consultation of geriatrician	2887 (90.6)	9184 (82.8)
Date of discharge	2843 (89.2)	9179 (82.8)
Complications*	2996 (98.5)	10,235 (96.4)
Mobility score	3000 (94.1)	9213 (83.1)
KATZ-ADL score	2989 (93.8)	10,176 (91.8)
Living situation	2902 (91.0)	8743 (78.9)
Completeness 3-month section, *n* (%)	*N* = 2847^‡^	*N* = 10,038^‡^
Follow-up section created^‡^	1246 (43.8)	3823 (38.1)
Reoperation^‡^	1104 (38.8)	2970 (29.6)
Mobility score^‡^	1059 (37.2)	3053 (30.4)
KATZ-ADL score^‡^	929 (32.6)	2727 (27.2)
Living situation^‡^	1053 (37.0)	2850 (28.4)

^∆^From April to December 2016

*These variables can only be recorded in the DHFA if indicated that an operation was performed; *N* = 3043 for 2016 and *N* = 10,612 for 2017

^¶^ER = emergency room

^‡^Includes only patients who were alive 4 months after surgery

### Time to surgery

The median time to operation for ASA 1–2 hip fracture patients was 18 h (IQR 7–23). Two hospitals performed significantly more operations within the median time of 18 h, and five hospitals performed significantly less operations within this time frame, with a variation between the hospitals of 29–75% (Fig. 1b). For ASA 3–4 hip fracture patients, the median time to operation was 21 h (IQR 13–27), with two hospitals operating significantly more patients within this time frame, while four hospitals operated significantly less patients within 21 h. The variation between the hospitals was 20–71% (Fig. 1c). Two hospitals had > 10% missing on the time to surgery variable of the ASA 1–2 patients and five hospitals of the ASA 3–4 patients and were therefore excluded from these analyses.

### Orthogeriatric management during admission

Orthogeriatric management during admission was provided to 78% of the operated patients aged 70 years and older. There was significant interhospital variation in the availability of comprehensive orthogeriatric management during admission, with 13 hospitals performing significantly better, and seven hospitals significantly worse than the mean (Fig. 1d). Orthogeriatric care was provided in a special comprehensive orthogeriatric ward in only 23% of the elderly patients. Six hospitals were identified as having a special comprehensive orthogeriatric ward, with four of these hospitals providing significantly more orthogeriatric management than the mean. (Fig. 1d).

Thirteen hospitals had > 10% of data missing on the variable orthogeriatric management and were excluded from these analyses.

## Discussion

This study describes the development and initiation of a nationwide hip fracture audit. Although the audit has not yet been implemented in all hip fracture operating hospitals in the Netherlands, and the participating hospitals do not yet register all of their patients, the audit already shows interhospital variation on the three quality indicators for hip fracture care that were studied. This variation can serve as a starting point for targeted interventions to improve the quality of hip fracture care in the Netherlands.

### Data completeness of the DHFA compared to other hip fracture audits

Two recent reviews identified other hip fracture audits, to which the data completeness in the DHFA can be compared [26, 27]. In its first full calendar year of registration, the DHFA achieved a nationwide case ascertainment of 58%. In the most recently published annual reports of other hip fracture audits, the case ascertainment ranged from 19 to 100% [3–9]. To the best of our knowledge, five hip fracture audits exceeded at this moment the 58% case ascertainment of the DHFA: Rikshöft in Sweden, the National Hip Fracture Database (NHFD) in the UK minus Scotland, the Danish Multidisciplinary Hip Fracture Registry (DMHFR), the Irish Hip Fracture Database (IHFD) and the Scottish Hip Fracture Audit (SHFA) [3–6, 8]. A possible explanation for the higher case ascertainment in these audits is that they are longer ongoing than the DHFA. The scores in the first and second years of the NHFD, which now has a 100% case ascertainment, are comparable to those of the DHFA. In the first and second NHFD years, respectively 20% and 56% of the patients were included [4, 28, 29]. The implementation of the NHFD improved when the Best Practice Tariff was introduced, a financial reward for hospitals meeting six targets [30, 31]. In the first full year of the patient level audit of the Australian and New Zealand Hip Fracture Registry (ANZHFR), 3519 patients were registered, which translates in a case ascertainment of approximately 14% [32]. In the second full year, this increased to 23% [9]. The coverage of the IHFD was better, with a case ascertainment of 78% in the first year and 84% in the second year [8, 33].

The average completeness of DHFA variables recorded after hospital discharge of 95% in the first year and 91% in the second year is comparable to that of other hip fracture audits. The NFHD had an average variable completeness of 92% in the first year and 98% in the second year and the IHFD 88% in the first year and 93% in the second year, while the ANZFR had a completeness of over 95% in both its first and second year [8, 9, 28, 29, 33]. The drop in the average variable completeness in the second year in the DHFA was also seen in the ANZFR [9]. A possible explanation is that in the second year of the DHFA, almost 2.5 times more patients were registered, which implies an increased risk of missing variables.

In 2017, the average completeness of variables recorded 3 months after operation was 30% in patients who were then still alive. In other hip fracture audits, the collection of follow-up data is difficult as well [34]. The ANZHFR accomplished a follow-up data collection rate of 50% in the fourth registration year (48% in Australia and 64% in New-Zealand) but had a low case ascertainment [9]. The NHFD had a 120-day follow-up percentage of 32% [4]. However, high follow-up rates are not beyond reach, as two hospitals in the NHFD managed to have follow-up data of 90% of the patients and the Scottish Hip Fracture Audit reported even a 120-day follow-up rate of 92% [35].

### Improving the data completeness of the DHFA

Since 2017, hospitals can use the DHFA to calculate and deliver the results of some of the mandatory national hip fracture quality indicators to two institutions that require this information: DNHI and HYCI. This may explain the high proportion (91%) of completed variables recorded at hospital discharge and the increase in case ascertainment to 58%. As of 2018, it is possible to deliver the results of all mandatory hip fracture quality indicators as demanded by the DNHI and HYCI through the DHFA. It is expected that this will further improve case ascertainment and data completeness in 2018. A financial reward, like the Best Practice Tariff for the NHFD, was and is not available for the DHFA [30, 31].

The operating hospital is responsible for retrieving and registering the data, both in-hospital and after discharge. But many hospitals do not see their patients back after discharge, unless a complication occurs during the recovery process which cannot be taken care of by, for example, a nursing home doctor. A possible solution to improve the 3-month follow-up data collection is to make this a joint responsibility of hospitals, nursing homes and home care organizations. The scientific committee of DHFA aims to establish an integrated transmural hip fracture care path in the Netherlands, with firmer integration of hospital care, nursing home care and home care. In this situation, the data is collected at the place where the patients are at the time of the intended follow-up moment. This integrated care would not only increase the number of patients registered in the DHFA but would also provide better insight in the overall quality of hip fracture care.

Comparison of the proportion of completed variables between hospitals provides insight into the data collection process. Hospitals where the data collection is well organized can serve as best practice for hospitals where this is not yet organized adequately.

### Differences in hip fracture care processes between hospitals

We observed significant differences in time to surgery and orthogeriatric management during admission between hospitals in the Netherlands. Other hip fracture audits have shown that these differences will reduce when feedback is provided to the hospitals about their performances [11, 13]. Farrow et al. also demonstrated with data from the Scottish Hip Fracture Audit that adherence to quality standards was associated with better patients outcomes [35].The average time to operation was 3 h longer for the ASA 3–4 group compared to the ASA 1–2 group. This was to be expected, since this patient category can benefit from optimization of their health status before surgery with a maximum delay up to 5 days [36]. More interesting is that five hospitals operated significantly fewer ASA 1–2 patients within the nationwide median time to operation, even though this group does generally not need to be optimized before surgery. As shown by the study of Hawkes et al., practice variance on time to surgery can be an incentive for an underperforming hospital to make targeted interventions to improve the time to operation [37]. However, the use of “timing of operation” as a quality indicator remains questionable [26].

The difference between hospitals in orthogeriatric management is interesting, as the national guideline states that every patient over 70 years should receive orthogeriatric management during admission [15]. Now only 78% of the patients above 70 receive orthogeriatric management during admission, which is low compared to the 2016 NHFD in which 89% of the patients above 60 years of age received orthogeriatric management [4]. A study with NHFD data also demonstrated that an increase of orthogeriatric treatment hours per patient was associated with a 3.4% relative risk reduction of mortality [38]. In the DHFA, only 23% of the patients is treated on a special ward with high orthogeriatrician hours per patient. Another recent study showed that a dedicated orthogeriatric ward lowered the 1-year mortality rate in frail elderly patients from 35.1 to 23.2% [39]. An additional analysis showed that patients receiving non-orthogeriatric treatment were significantly younger and had less comorbidities. It will be interesting to evaluate the effects of non-orthogeriatric treatment on the outcomes of care for this specific population. The data from the DHFA enables such a study.

In the start-up phase of the DHFA, hospitals will be compared on process of care only. This will provide hospitals the opportunity to first optimize their hip fracture care process. Later, hospital performances will be compared on outcomes of care.

### International benchmarking

In addition to benchmarking hospitals in the Netherlands, an audit can also provide insight into how treatment patterns differ between countries [40]. To enable international benchmarking, Sáez-López et al. compared the content of existing hip fracture audits and proposed variables which should be collected in a hip fracture registry; almost all of the proposed variables are collected by the DHFA [27]. In line with Sáez-López et al. and Johansan et al., case mix and treatment characteristics of different nationwide hip fracture registries were compared (see Table 2) [27, 41]. The DHFA seems comparable with other nationwide hip fracture audits in terms of case mix, as the common Dutch hip fracture patient is a female above 80 years of age with an ASA score of 3 or higher. Compared to the other audits, in the Netherlands, intramedullary fixation is used more often, whereas sliding hip screws are used less frequently. The Dutch guideline for proximal femoral fracture does not include definite recommendations as to the type of osteosynthesis to be used in case of a pertrochanteric femur fracture (31—A1/31—A2/31—A3). It is up to the local protocol or surgeon to decide which type of osteosynthesis will be used. Apparently, there is a preference in the Netherlands for using intramedullary fixation, since 73% of the type 31—A1 fractures were treated in this way. This finding can serve as a starting point for further outcome studies to explain whether and how differences in treatment relate to differences in outcome of care.

**Table 2:** Comparison of implementation and patient characteristics in eight nationwide hip fracture audits

	Rikshöft	SHFA	NHFR	NHFD	IHFR	ANZHFR		DMHFR	DHFA
Country of audit	Sweden	Scotland	Norway	UK	Ireland	Australia	New Zealand	Denmark	The Netherlands
Year audit started	1988	1993–2008, restart 2015	2005	2007	2012	2013		2013	2016
Included number of patients in 2017	15,062*	3942	8422	65,645	3159	5178	730	6679	11,086
Yearly estimated number of hip fractures	18,000	6000	–	65,645	3650	22,000	3803	6679	19,000
Age for inclusion in audit	15	50	–	60	60	50	50	65	18
Average or median age in years	82	82	83	83*	81^¶^	82	83	83	82
Female (%)	67	73	70	72∆	69	70	68	69	67
ASA score (%)									
I	–	–	3	2∆	2	2	1	–	6
II	39*◊	26*◊	32	25∆	39	20	22	–	30
III	53*	53*	56	54∆	53	56	56	–	44
IV–V	8*	15*	8	14∆	7	22	20	–	5
Unknown/missing	–	–	1	4∆	–	–	–	–	15
Fracture type (%)									
Femoral neck non-dislocated	13	17*	13	9	9	17*	15*	10*	14
Femoral neck dislocated	39	36*	42	49	43	29*	37*	45*	32
Intertrochanteric	37	38*	30	32	36	46*	43*	37*	33
Subtrochanteric	8	4*	6	6	7	8*	5*	7*	2
Other/unknown/missing	3	5*	9	4	6	0*	0*	1*	19
Type of anaesthesia (%)									
General anaesthesia	5*	50*	10	41*	11	70*	56*	–	30
Spinal anaesthesia	95*	44*	86	50*	58	27*	41*	–	45
Regional anaesthesia	–	–	–	–	–	3*	3*	–	1
Other/missing	–	6*	4	9*	28	–	–	–	24ˆ
Fracture treatment (%)									
Conservative	–	–	–	2	–	–	–	–	2
Cannulated hip screw	17*	2*	14	3	2	4*	13*	10*	6
Sliding hip screw	22*	36*	22	32	25	19*	22*	22*	13
Intramedullary fixation	27*	7*	17	12	21	36*	30*	31*	38
Hemiarthroplasty	25*	44*	41	43	45	33*	26*	25*	34
Total hip replacement	9*	6*	4	8	3	8*	9*	10*	5
Other/unknown/missing	–	–	2	0	2	–	–	–	1
Mobility before fracture (%)									
Freely mobile without aids	43*	50	–	36.4∆	46	47	43	–	37
Mobile outdoors with one aid	–	17	–	21.9∆	–	12	11	–	5
Mobile outdoors with two aids or frame	–	22	–	14.8∆	–	36	35	–	26
Some indoor mobility but never goes outside without help	–	10	–	23.7∆	14	–	–	–	6
No functional mobility (using lower limbs)	–	1.4	–	1.7∆	2	2	2	–	2
Unknown/missing	–	–	–	1.5∆	–	3	7		24
Living situation before fracture (%)									
Living independently at home	70*	75	–	81	81	71	76	73*	44
Living independently with help of activities of daily	–	–	–	–	–			–	16
Home care	26*	18	–	11	–	28	24	19*	7
Nursing home	–	–	–	8	9	–	–	–	10
Nursing home with rehabilitation	–	–	–	–	–	–	–	–	1
Different living situation	–	–	–	–	9	–	–	–	2
Unknown/missing	–	–	–	–	–	–	–	–	21

Source is 2017 annual report of audit; if 2017 annual report was not available, the 2016 annual report was taken. The year of the annual report should be placed after the full audit name.

Rikshöft (Sweden) 2016, SHFA = Scottish Hip Fracture Audit 2017, NHFR = Norwegian Hip Fracture Register 2017, NHFD = National Hip Fracture Database 2017 (United Kingdom minus Scotland), Irish Hip Fracture Audit 2016, ANZHFR = Australian and New Zealand Hip Fracture Registry 2017, DMHFR = Danish Multidisciplinary Hip Fracture Registry 2017, DHFA= Dutch Hip Fracture Audit 2017.

*Source: [41]

∆Source: NHFD annual report 2016

◊ASA I and ASA II together

¶Average age is 79 for men and 81 for women

^Other anaesthesia in the DHFA is general and regional anaesthesia (2%), general and spinal anaesthesia (1.5%), spinal and regional anaesthesia (4.9%), and missing (15%)

### Limitation

A limitation of the present study is the fact that the DHFA has a current national audit case ascertainment of 58%. This percentage implies a possible bias in the audit population, as a certain population may not be included in the registration. However, in our opinion, the missing patients are missing completely at random, the underlying reasons being most likely a lack of staffing capacity for data collection and the fact that not all hospitals participate in the DHFA at present. Benchmarking hospitals is possible, but establishing differences between hospitals with low numbers of inclusion is difficult as they provide wide confidence intervals.

Another limitation could be the accuracy of the data. Two studies showed that data in hip fracture audits were sometimes incorrectly registered, and that it is important that entered data is validated [42, 43]. We believe when the data verification is directly done in the web-based survey, and when external data verification is performed every 3 years, that the registered data can be considered accurate.

Hospitals are required by law to report their results on quality indicators to the DNHI and HYCI every calendar year. To ensure more objective and reliable data, the DHFA can be used to deliver the mandatory quality indicator results to the DNHI and HYCI, but the use of the DHFA is not obligatory. As shown by another audit, obligatory data delivery leads to full participation [20].

## Conclusion

Two years after the implementation of the DHFA, not all hospitals participate in the audit, and the data gathering process within participating hospitals needs to be further optimized.

Based on the results so far, there seems to be considerable practice variance between hospitals in the Netherlands concerning both time to surgery and orthogeriatric management. These differences illustrate the need for further development and implementation of the DHFA and provide potential starting points for improvements. The next step is achieving a higher case ascertainment so that hospitals can be benchmarked on outcomes of care and quality of care can be improved.

## Appendix

**Table 3:** Dutch Hip Fracture Audit dataset

*General patient information*	
Country	
Citizen service number	
Name	
Sex	Male Female
Date of birth	Date
Disease date (if available)	Date
Name Hospital	
Admission *Emergency department*	
Head practitioner	Trauma surgeon Surgeon Orthopaedic trauma surgeon Orthopaedic surgeon Geriatrician Internist-elderly Internist Nursing home doctor
Fellow practitioner	Trauma surgeon Surgeon Orthopaedic trauma surgeon Orthopaedic surgeon Geriatrician Internist-elderly Internist Nursing home doctor
Date and time of admission to the emergency department	Date
Date and time of leaving the emergency department	Date
Living situation before fracture	Living independently at home Living independently with help of activities of daily Home care Nursing home Nursing home with rehabilitation Different living situation Else
Type of involved of geriatrician / Internist-elderly	None Post-operative consultation Peri-operative consultation Treatment on ortho-geriatric ward
Dementia	No Yes Unknown
Medication for osteoporosis	No Yes
Pre-fracture Mobility Score	Freely mobile without aids Mobile outdoors with one aid Mobile outdoors with two aids or frame Some indoor mobility but never goes outside without help No functional mobility (using lower limbs) Unknown
KATZ-6 ADL	Bathing? Independence / Dependence Dressing? Independence / Dependence Toileting? Independence / Dependence Continence? Independence / Dependence Transferring? Independence / Dependence Feeding? Independence / Dependence
SNAQ score	Did you lose weight unintentionally? More than 6 kg in the last 6 months More than 3 kg in the last month Did you experience a decreased appetite over the last month? No/Yes Did you use supplemental drinks or tube feeding over the last month? No/yes
Admission *Operation*	
Type of fracture	Femoral neck non-dislocated Femoral neck dislocated Intertrochanteric AO – A1/A2/A3 Subtrochanteric Not specified
Fracture Treatment	Conservative Cannulated hip screw Sliding hip screw Intramedullary fixation Hemiarthroplasty Total hip replacement
Date and time of operation	Date
Side of fracture	Right Left Both
Bone graft used	No Yes
ASA-score	I II III IV V
General anaesthesia	No Yes
Spinal anaesthesia	No Yes
Regional anaesthesia	No Yes
Admission *Complications during admission*	
Complication	No Yes
	If yes; Anemia Stroke Chronic Obstructive Pulmonary Disease Pressure ulcer Heart failure Delirium Deep vein thrombosis Dislocation implant Electrolyte disorder epilepsy Fracture around prosthesis Phlebitis Hematoma Pulmonary embolism Loosening of fixation material Heart infarct Kidney failure Pneumonia Cardiac arrhythmia Urinary tract infection Fall Superficial wound infection Deep wound infection Other complication
Admission *Discharge*	
Died during hospital stay	No Yes Unknown
Date of discharge	Date
Mobility at discharge	Freely mobile without aids Mobile outdoors with one aid Mobile outdoors with two aids or frame Some indoor mobility but never goes outside without help No functional mobility (using lower limbs) Unknown
Osteoporosis medicine at discharge	No Yes
Living situation after discharge	Living independently at home Living independently with help of activities of daily Home care Nursing home Nursing home with rehabilitation Different living situation Else
*Follow-up* *3 months after operation*	
Date of follow-up	Date
Side of fracture	Right Left Both
Reoperation within 60 days	No Yes Unknown
	If yes: Date of reoperation.
Died before follow-up	No Yes
Living situation after 3 months	Living independently at home Living independently with help of activities of daily Home care Nursing home Nursing home with rehabilitation Different living situation Else
Mobility after 3 months	Freely mobile without aids Mobile outdoors with one aid Mobile outdoors with two aids or frame Some indoor mobility but never goes outside without help No functional mobility (using lower limbs)
KATZ-6 ADL after 3 months	Bathing? Independence / Dependence Dressing? Independence / Dependence Toileting? Independence / Dependence Continence? Independence / Dependence Transferring? Independence / Dependence Feeding? Independence / Dependence
*Follow-up* *1 year after operation*	
Date of follow-up	Date
Side of fracture	Right Left Both
Died before follow-up	No Yes

**Table 4:** Definition of quality indicators

1. Variable completeness	
Operationalization	The proportion of completed variables per hospital.
Numerator	The number of variables that are completed per patient.
Denominator	The total number of eligible variables per patient.
Definition	Variables used: date of birth, gender, type of fracture, type of treatment, ASA score, date and time of arrival at emergency department, date and time of surgery, consultation of geriatrician, date of discharge, type of anaesthesia, complications, Katz Index of Independence in Activities of Daily Living at admission, mobility score at admission, living situation before admission, reoperations, 3-month Katz Index of Independence in Activities of Daily Living at admission, 3-month mobility score, 3-month living situation.
Inclusion criteria	Patients older than 18 years with hip fracture who received an operative treatment.
Exclusion criteria	Patients not eligible for 3-month follow-up.
Inclusion period	1 January 2017–31 December 2017
2. Time to surgery	
Operationalization	Median time to surgery from admission to the emergency department and start of surgery of ASA 1/2 and ASA 3/4 patients with a hip fracture.
Numerator	a. The number of ASA 1/2 patients operated within the audit median time difference in hours from admission to start of operation. b. The number of ASA 3/4 patients operated within the audit median time difference in hours from admission to start of operation.
Denominator	a. The total number of ASA 1/2 patients operated. b. The total number of ASA 3/4 patients operated.
Definition	–
Inclusion criteria	Patients older than 18 years with hip fracture who received an operative treatment.
Exclusion criteria	A time to surgery of > 120 h is defined as missing; hospitals with > 10% missing on the counter are excluded.
Inclusion period	1 January 2017–31 December 2017
3. Orthogeriatric management during admission	
Operationalization	Orthogeriatric management during admission for operated hip fracture patients.
Numerator	Number of patients with orthogeriatric treatment during admission.
Denominator	Total number of operated hip fracture patients older than 70 years.
Definition	Orthogeriatric treatment: peri-operative collaboration between geriatrician and surgeon.
Inclusion criteria	Patients 7o years and older with a hip fracture who received an operative treatment.
Exclusion criteria	Missing data is treated as no orthogeriatric management; hospitals with > 10% missing on the counter are excluded.
Inclusion period	1 January 2017–31 December 2017

## Abbreviations

*DHFA*: Dutch Hip Fracture Audit
*DNHI*: Dutch National Healthcare Institute
*HYCI*: Health and Youth Care Inspectorate
*DICA*: Dutch Institute for Clinical Auditing
